# Influence of Association

**Published:** 1861-08

**Authors:** J. G. Hamill


### INFLUENCE OF ASSOCIATION.

BY DR. J. G. HAMILL.

Read before the Central Ohio Dental Association, at Newark, July 10th, 1861.

*Gentlemen of the Central Ohio Dental Association*:—Almost every section of our State has, for some time past, had its professional altar erected; and lit up, too, by the beams of associated effort.

The light thus sent out has been the means of illuminating many obscure recesses, and bringing thence the hidden treasures of many diffident and careless individualities.

It is a matter of regret to me, as well as to yourselves, that the profession of this central region of Ohio has blundered along for so many years without having erected an altar to the same divinity. We have received blessings without number, yet not in answer to our own invocations; but merely through the merciful dispensations of *Him*, who rains blessings both upon the just and unjust. Truly may it be said, that if we have been worshiping anything at all professionally, it has been the "unknown god." Individual efforts, for bringing about a better state of things in this section, have not been wanting; but what, pray tell me, can such efforts accomplish in the face of exceedingly selfish cotemporaries, and a community much more learned in the practice of parsimony, than in a knowledge of the benefits arising from a correct and professional course of dental practice? Under such circumstances, individual effort can only accomplish about enough to thoroughly disgust a man that feels genuine professionul promptings. We must, therefore, have a union of hands, of heads and of hearts, in order to insure the respect of learned men in all the other professions, as well as to dispel forever that crushing manifestation of parsimony, which community persists in forcing upon us.

We have, doubtless, frittered away many golden privileges by not uniting in associated effort long ago. But I hope for better things in the future; and your presence here I take as actual confirmation of my hopes. I do not expect to see much accomplished at this meeting, because the maddening rush of "grim-visaged war" is upon us, and the mind has grown sick at contemplation of anything except that which tends to crushing rebellion, and redressing the wrongs of an insulted nationality. But while our brothers, sons and friends challenge the cannon's mouth for God and Liberty, let us not altogether forget that our commissions have not yet expired; that we are still called upon to fight against everything that throws its ugly carcass into our ranks, for the purpose of lowering the standard of our profession. The ills of humanity demand that we not only support that standard in its present position, but raise it higher, and still higher, until perfection echoes back relief to every torturing ill that flesh is heir to.

We have come together, then, to do battle for the cause of truth and progress, and God grant that there may be no element of discord among us—that this society, founded as it has been on a basis broad and liberal, may have a superstructure joined together by the *ties* of our better natures.

You all know that one of the most prominent principles of animal life is self-preservation—(or in more common acceptation)—*selfishness.* We are furnished with the most incontestible evidence of this, whenever or wherever we look upon animated nature. The dog, however much he may love his master, does not hesitate to steal his meat, but is foolish enough even to plunge into the water, which by chance may be under him, after the very shadow of more. You may strike a hog with an ear of corn as hard as you are able—he will grunt, snatch up the corn in his mouth and run. You may throw feed to a lion, and if you do not glut him to sleepiness, he will turn and rend the life out of you. Such is animal life with nothing but instinct and physical force to direct and accomplish the aims of existence.

Man, uneducated, is an animal. He does not possess the characteristic of humanity. He has not the element within him, which alone is able to direct and hold in subjection the promptings of animal nature. Selfishness is the ruling passion of *his* nature, as it is of all other animals. In a word, he is a cannibal, and manifests his love for his fellow beings by eating them. But he leaves this mere brute life, and ascends in the scale of humanity, just in proportion as the mind is developed, and he is enabled thereby to judge between right and wrong. The rigor of the law and the usages of society are the means that direct and control him after he is able to judge thus, but has not the will power to enable him at once to practice the former and discard the latter. We are all possessed of this attribute of selfishness, and the use *we* make of it is regulated either by that degree of education which appeals solely to the law and the established usages of society, or by a higher law, which is prompted by reason and the affections. These laws are as different, the one from the other, as is right from wrong. To the transgressor of the former, a penalty in exact keeping with the heinousness of his crime, is meted out. To the transgressor of the latter there is no penalty attached, except the occasional twinge of a half-awakened conscience. To the observance of the former there is no reward; but for the observance of the latter there is the reward of an approving conscience, and the enjoyment of the society of the good and great. In fact, it is the passing over from this mere intellectual assent to the right of a thing, to the voluntary espousal of it by the affections, which makes a man good and great. This crucible into which the intellect and the affections are melted down together, we recognize in the avocations of life as *honor*. All the avenues of life claim to have some of this in their structure ; but the professions build upon it most largely. In fact, it is the only rock upon which any man can with certainty build his hopes of reaching the honors and emoluments of any profession.

All of us desire to occupy a higher position in *our* profession than we are now occupying, and this is the manifestation of a laudable selfishness. How to attain such a position, "that's the question." On its solution hangs our own happiness and usefulness, as well as the usefulness and happiness of many others. That which, more than anything besides, restrains and keeps our actions within the bounds of professional courtesy, while we are endeavoring to advance our interests and well-being, is personal intercourse with our cotemporaries. By seeing their faults, we may be led to see that we have some ourselves, that need correction. The youth, without experience, and with extravagant aspirations, goes off in a dream of gaining riches suddenly, by which he expects to unlock the very gates of Paradise. This unfits him altogether for the performance of the common routine of practical life. He awakes from this dream some day, and hastily concludes that he must go up by virtue of the downfall of one or more of his cotemporaries. And so he gets up a war about patents or modes of practice, by which his interests suffer, and he is thought by community to be meddlesome. Later in life he comes to the conclusion that he has not ascended the hill of science so fast as his friend across the way, that he tried to pull down. He "tacks about" and shapes his course in accordance with the lessons experience has taught him. Still later in life, though he may be "full of wise saws and modern instances," he *knows* that the journey of life that leads to distinction is one involving years of straight-forward, honest, constant labor. The dream of youth has long since passed away, and the airy castles he built have vanished into *thin* air. The passion, too, which led him to the belief that the downfall of others was the key to his own exaltation, has given way, and become softened by the teachings of *current events* which have transpired with and about him. In a word, his selfishness has become educated—and educated selfishness does not, like a dog, flee from the substance to catch its shadow. It does not, like the hog, submit to ignominy, that it may snatch the wealth an educated man spurns from him when he feels his "honor grip." Neither will it, like the wild beast, receive all a generous hand will give it, and then *take* what remains, even to the death. No, it is willing to let live, as well as live. In fact, selfishness, when controlled and directed by education and the affections, we can recognize as nothing less than the chief of Christian virtues—charity. Alas!—that it is one of the things in this world so "slowly learnt."

That condition of any community, society or profession which is characterized by a stable, equitable and liberal government, is always marked by a highly cultivated state of mind, a gentlemanly bearing and reciprocal regard for the feelings and interests of associates and cotemporaries. You can not, therefore, fail to appreciate the value of the two leading principles of action incorporated into the preamble to the constitution of this society. The benefits arising from the first of these (professional education) you must acknowledge. The personal experience, resulting from the labor of many years, or observation of the prosperity attending those, although very young, that have enjoyed advantages for acquiring professional education, which have been denied to you, or perhaps which you have denied to yourself, must teach you to recognize its power. The value of the second (that of personal intercourse) we now have a chance of testing. It will be valuable just in proportion as we are enabled to make it the vehicle of communicating knowledge to each other while here, and of reciprocating kindly feelings and actions toward each other after we return to our several individual fields of labor.

There was a time when a dentist was excusable for not being a professional man; but that time has now subsided. There was a time when dentistry did not possess the requisites which go to make up a profession. That was when artificial teeth were not fit for use in the process of mastication, but were worn merely for "*looks' sake.*" And oh! what a figure they cut, too, on that score. Afterwards they became practicable, and then *the trade* was established. And I imagine that if all the ghosts of the teeth that have been sacrificed to the importunities of the *trade* were now brought up and arrayed before us, we would not only feel mortified at the sight; but we would also feel that a long life of educated, conservative dental labor would be but small return for such heathenish practice.

But the *trade*, through the instrumentality of dental colleges, the press, and associations, has been transformed into the profession; and now he who avails himself of these channels for the acquisition of knowledge, does not often suffer the mortification of having to extract comparatively good teeth, for the want of a knowledge of their proper treatment.

Dental colleges claim our attention first, because they mold the student to fixed principles of action, which carry him through the avenues of true professional life. To young men, college advantages are beyond calculation, because they enable him to start in practice on equal footing with older practitioners, that have not availed themselves of a like preparation. Such young men as avail themselves of the advantages which our colleges afford, are not obliged to "cast their bread upon the waters, and await its return after many days;" but they realize its return at once. There is no unfolding of humanity, I think, so grand as that which comes forth in youth, bearing with it full evidence of the moral, social and legal security for the individual, and the profession he repre sents.

Dental colleges give this security to every man that passes through them, in accordance with their established rules ; and it is but seldom such security is not accepted and requited, on the part of community, by a return of liberal patronage. But I have an especial duty to perform in this connection, which I must not shrink from. That duty is to call your attention to the claims of the Ohio Dental College upon this society. I was deeply mortified that the complaint of a want of patronage had to go forth from the lips of one of the Faculty at the last Commencement of that college. I felt the justice of the accusation, and the alumni of the college everywhere *ought to feel it.* I intend to speak of it here, and act on it elsewhere to the best of my ability, and I hope you will all *act* also. There are about forty or fifty dentists in this section of the State, that ought to be members of this society. As far as I have been able to learn, but fifteen of these are graduates of any college, and only about half of these of the Ohio college. If I am correctly informed, these graduates, in most instances, do the leading business in their respective localities, and the reason they don't do so in all, is simply because they compete with men who have spent half a lifetime in the practice. Why the young men that are constantly coming into the profession can't see and profit by these things is more than I can tell. Yet they continue to enter the profession by the back door, and drag along for years, a mere bundle of complaints about the want of patronage, and the cold, unsympathizing world, before they are able to make good the claims they have proffered. Better, far better would it be for such to make all sorts of sacrifices at the outstart, in order to enter the profession through the correct channels. Then a little close attention to office duties will bring about the honors and emoluments of appreciated professional worth. Whose fault it is that young men enter the profession so carelessly, is a matter of *controversy.* I hope those that are in the habit of taking students for six or nine months can wash their hands clean of it. Some dentists complain of their students opening offices near by them, and taking their practice by the inducements they offer in the way of low prices. Will such *complainants* allow me to recommend thorough professional education as a remedy for such occurrences? That student that is able to do as good work as his preceptor, is not going to be fool enough to do it for any less compensation.

The "press" is the next great means for acquiring knowledge. And when we look back, and observe how rapidly the number of its issues has increased within a few years, we must conclude that the profession appreciate its power. But I believe our journals have not the support of more than one-half of those that claim to be of the profession. This ought not to be so, and there is no valid reason why it should be so. I venture the assertion, and I do it too in all kindness, that two-thirds of those who do not take any dental publication, plead poverty as the reason for not doing it,—plead it, too, in the very face of the fact that they spend more money every year for *lager*, *brandy smashes* and tobacco, than would pay the subscription for the four leading dental journals of America. These are facts which any of you may find out for yourselves by a very little observation. And what, my friends, must we conclude from such facts? Of necessity we are forced to think that such individuals ride the profession as a hobby merely, for the gratification of their own selfish appetites, losing sight entirely of the high trust they have in keeping, which, as accountable beings, they are bound to extend and perfect for the alleviation of suffering humanity.

And now we have only to consider the last of the attributes of this triune sovereign to progress and distinction—association. And although it is the least of these, yet like its prototype on the road to the Christian's life, it is the means of awakening the *sinner*—it is the means of arousing the minds of men to the necessity of occupying a higher sphere in professional life, and of enabling them to see, and lay hold of the facilities for the attainment of such an object.

About twenty years ago, the first rays of light brought to view by associated effort, came from the American Society of Dental Surgeons, through its organ, the American Journal. These rays illuminated the understanding ef leading men, and soon after the Baltimore College stood forth, the first of its kind on earth. Without pausing to note the rise and fall of other societies of less note, we come to the M. V. Dental Association (now the oldest in existence), which was organized about five years after that just mentioned. Its doctrines were heralded through the land by *its* adjunct power, the Dental Register, and very soon after, the Ohio Dental College was established.

We can hardly realize how quickly these events followed each other. It appears almost like the flash of chemical action. The elements were thrown together by association, a union took place, resulting in a compound, capable of healing up the fetid sores of charlatanism, by which the professional body had been afflicted so long and grievously.

My friends, I wish to see this awakening influence which association breathes infused into us, and also all the dentists resident within the contemplated jurisdiction of this society. We do not propose to establish journals and found colleges; but we wish to elevate ourselves and our profession, and we know of no way of accomplishing this so efficiently as by supporting the journals now established, and the colleges already founded. If we do this, the personal respect due to the interests and feelings of each other in our respective fields of labor will necessarily follow, placing us in a position for "dwelling in unity together."

